# Nitric oxide protects against HIV gp120 endothelial injury

**DOI:** 10.1186/1758-2652-13-S4-P68

**Published:** 2010-11-08

**Authors:** S Fiorucci, A Mencarelli, D Francisci, E Distrutti, B Renga, F Baldelli

**Affiliations:** 1 University of Perugia, Infectious Diseases Clinic, 1 Department of Clinical and Experimental Medicine, Perugia, Italy; 2 Ospedale S.Maria della Misericordia, 1 Department of Clinical and Experimental Medicine, Perugia, Italy

## Purpose

The SMART study demonstrated that an higher number of cardiovascular events had occurred in patients receiving an intermittent therapy, suggesting HIV itself could contribute to the increased cardiovascular risk. Moreover vascular dysfunctions in HIV-1 patients have long been documented before era HAART. In addition to an indirect damage caused by pro-inflammatory cytokines, it is increasingly recognized the HIV infection might injury endothelial cells by both a direct damaging activity linked to cell invasion and virus replication and an indirect effect depending on viral proteins, such as TAT and gp120. Previous studies have demonstrated NO effectively protects endothelial cells from apoptosis and inflammation caused by a variety of noxious agents, but its efficacy against gp120 injury is unknown. In order to achieve new insights in NO activity in the endothelial injury by HIV we have investigated whether, in an in vitro model, NO ± aspirin protects against endothelial apoptosis induced by the HIV gp120*.*

## Methods

Human umbilical cells (HUVEC) supplemented with human epithelial growth factor were used. Cells were cultured with 100 µM DETA-NO alone or in combination with aspirin 100 µM, in the presence of GP-120 1 µg/ml. The detection of nitrite/nitrate, mitochondrial membrane potential, the cytochrome c release into the cytosol and of caspase activities were performed.

## Results

We observed:

1) HIV-1 gp120 has apoptotic activities in HUVEC cells.

2) DETA-NO, which slowly and continuously release NO, decreases the induced apoptosis induced by gp 120.

3) HIV-1 gp120 causes mitochondrial depolarization, releases of cytochrome c into the cytosol and increases caspase 9 activity.

4) NO prevents the gp120-induced mitochondrial depolarization and protects against cytochrome c translocation into the cytosol and the increased caspase activity.

5) These protective activities were not reproduced by aspirin.

## Conclusions

These data provide further support the notion the HIV itself might promote endothelial injury and lead to increased cardiovascular complications. Moreover, this study grounds the basis to development of NO-based strategies for cardiovascular protection of HIV infected persons.

